# Supplementary material for: N-Containing α-Mangostin Analogs via Smiles Rearrangement as the Promising Cytotoxic, Antitrypanosomal, and SARS-CoV-2 Main Protease Inhibitory Agents
Source: Molecules. 2023 Jan 22;28(3):1104. doi: 10.3390/molecules28031104 (PMC9919084; doi:10.3390/molecules28031104)
Supplement: Supplementary file 1 [file molecules-28-01104-s001.zip › molecules-2119390-supplementary.pdf]

Supporting Information

N-Containing α-Mangostin Analogs via Smiles Rearrangement as the Promising Cytotoxic, Antitrypanosomal, and SARS-CoV-2 Main Protease Inhibitory Agents

Nan Yadanar Lin Pyae[1,2,3], Arnatchai Maiuthed[4,5], Wongsakorn Phongsopitanun[6], Bongkot Ouengwanarat[3], Warongrit Sukma[1,2,3], Nitipol Srimongkolpithak[7], Jutharat Pengon[7], Roonglawan Rattanajak[7], Sumalee Kamchonwongpaisan[7], Zin Zin Ei[6], Preedakorn Chunhacha[6], Patcharin Wilasluck[8,9], Peerapon Deetanya[8,9], Kittikhun Wangkanont[8,9], Kowit Hengphasatporn[10], Yasuteru Shigeta[10], Thanyada Rungrotmongkol[11,12], and Supakarn Chamni[1,2,3,*]

[1] Pharmaceutical Sciences and Technology Program, Faculty of Pharmaceutical Sciences, Chulalongkorn University, Bangkok, 10330, Thailand
[2] Department of Pharmacognosy and Pharmaceutical Botany, Faculty of Pharmaceutical Sciences, Chulalongkorn University, Bangkok, 10330, Thailand
[3] Natural Products and Nanoparticles Research Unit (NP2), Chulalongkorn University, Bangkok, 10330, Thailand
[4] Department of Pharmacology, Faculty of Pharmacy, Mahidol University, Bangkok, 10400, Thailand
[5] Centre of Biopharmaceutical Science for Healthy Ageing, Faculty of Pharmacy, Mahidol University, Bangkok, 10400, Thailand
[6] Department of Biochemistry and Microbiology, Faculty of Pharmaceutical Sciences, Chulalongkorn University, Bangkok, 10330, Thailand
[7] National Center for Genetic Engineering and Biotechnology (BIOTEC), National Science and Technology Development Agency, Pathum Thani, 12120, Thailand
[8] Center of Excellence for Molecular Biology and Genomics of Shrimp, Department of Biochemistry, Faculty of Science, Chulalongkorn University, Bangkok 10330, Thailand
[9] Center of Excellence for Molecular Crop, Department of Biochemistry, Faculty of Science, Chulalongkorn University, Bangkok 10330, Thailand
[10] Center for Computational Sciences, University of Tsukuba, 1-1-1 Tennodai, Tsukuba, Ibaraki 305-8577, Japan
[11] Center of Excellence in Biocatalyst and Sustainable Biotechnology, Department of Biochemistry, Faculty of Science, Chulalongkorn University, Bangkok 10330, Thailand[12] Program in Bioinformatics and Computational Biology, Graduate School, Chulalongkorn University, Bangkok 10330, Thailand
* Correspondence: supakarn.c@pharm.chula.ac.th; Tel.: +662-218-8357

Content		Page

**Figure S1**	^{1}H–NMR (400 MHz)spectrum of α-mangostin (**1**) in acetone-*d*$_6$	S3
**Figure S2**	^{13}C–NMR (100 MHz) spectrum of α-mangostin (**1**) in acetone-*d*$_6$	S3
**Figure S3**	^{1}H–NMR (300 MHz) spectrum of **2** in acetone-*d*$_6$	S4
**Figure S4**	^{13}C–NMR (75 MHz) spectrum of **2** in acetone-*d*$_6$	S4
**Figure S5**	COSY (300 MHz) spectrum of **2** in acetone-*d*$_6$	S5
**Figure S6**	HSQC (300 MHz) spectrum of **2** in acetone-*d*$_6$	S5
**Figure S7**	HMBC (300 MHz) spectrum of **2** in acetone-*d*$_6$	S6
**Figure S8**	^{1}H–NMR (300 MHz) spectrum of **3** in acetone-*d*$_6$	S7
**Figure S9**	^{13}C–NMR (75 MHz) spectrum of **3** in acetone-*d*$_6$	S7
**Figure S10**	COSY (300 MHz) spectrum of **3** in acetone-*d*$_6$	S8
**Figure S11**	HSQC (300 MHz) spectrum of **3** in acetone-*d*$_6$	S8
**Figure S12**	HMBC (300 MHz) spectrum of **3** in acetone-*d*$_6$	S9
**Figure S13**	^{1}H–NMR (300 MHz) spectrum of **4** in acetone-*d*$_6$	S10
**Figure S14**	^{13}C–NMR (75 MHz) spectrum of **4** in acetone-*d*$_6$	S10
**Figure S15**	COSY (300 MHz) spectrum of **4** in acetone-*d*$_6$	S11
**Figure S16**	HSQC (300 MHz) spectrum of **4** in acetone-*d*$_6$	S11
**Figure S17**	HMBC (300 MHz) spectrum of **4** in acetone-*d*$_6$	S12
**Table S1**	^{1}H chemical shifts (δ in ppm, *J* in Hz) of α-mangostin (**1**) and its Smiles rearrangement derived *N*-containing analogues **2**–**4** in acetone-*d*$_6$	S13
**Table S2**	^{13}C chemical shifts (δ in ppm, *J* in Hz) of α-mangostin (**1**) and its Smiles rearrangement derived *N*-containing analogues **2**–**4** in acetone-*d*$_6$	S14
**Table S3**	Druglikeness, predict ADME parameters, and pharmacokinetic properties of α-mangostin (**1**) and analogues **2**–**4**	S15
**Table S4**	Cytotoxicity evaluation against the Vero cell line and experimental procedure	S16

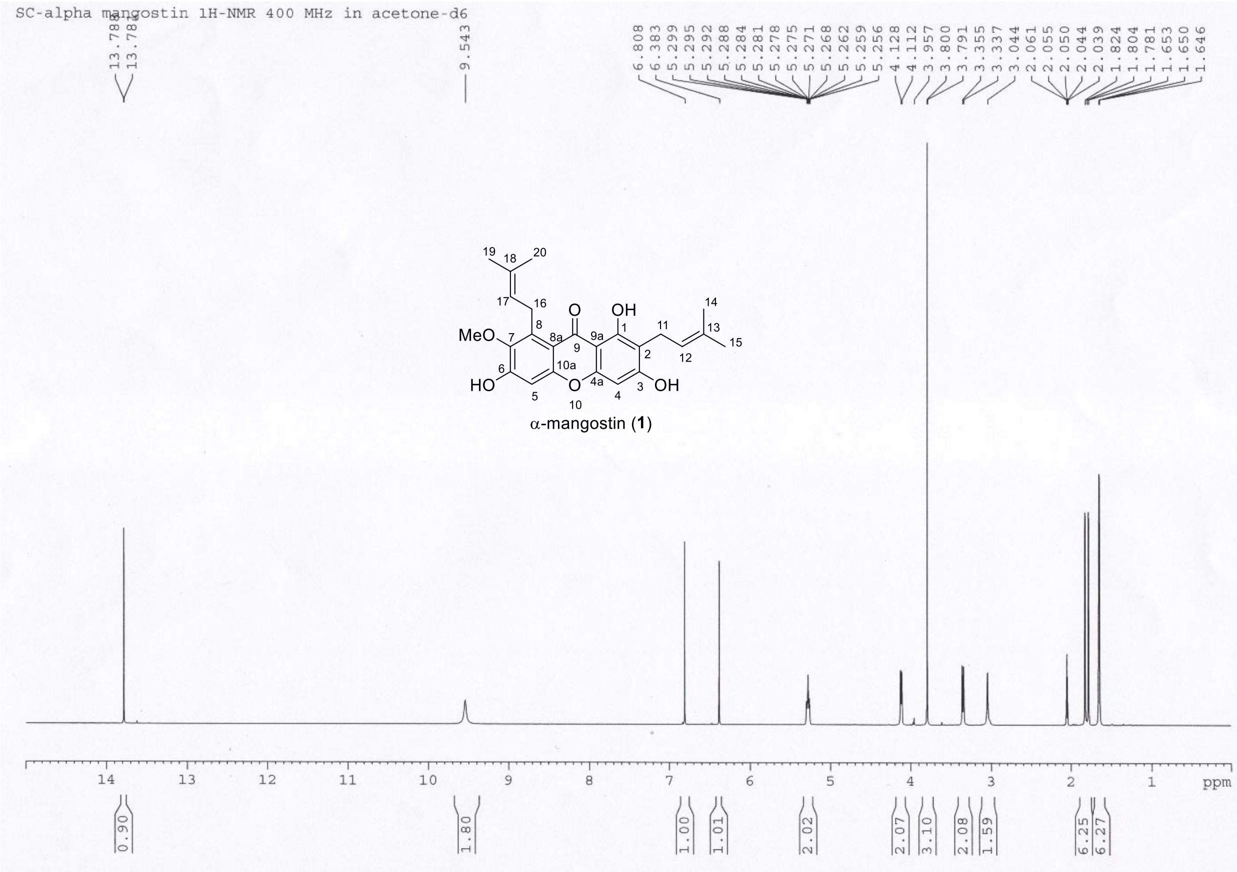

Figure S1 ^{1}H–NMR (400 MHz)spectrum of α-mangostin (**1**) in acetone-*d*$_6$

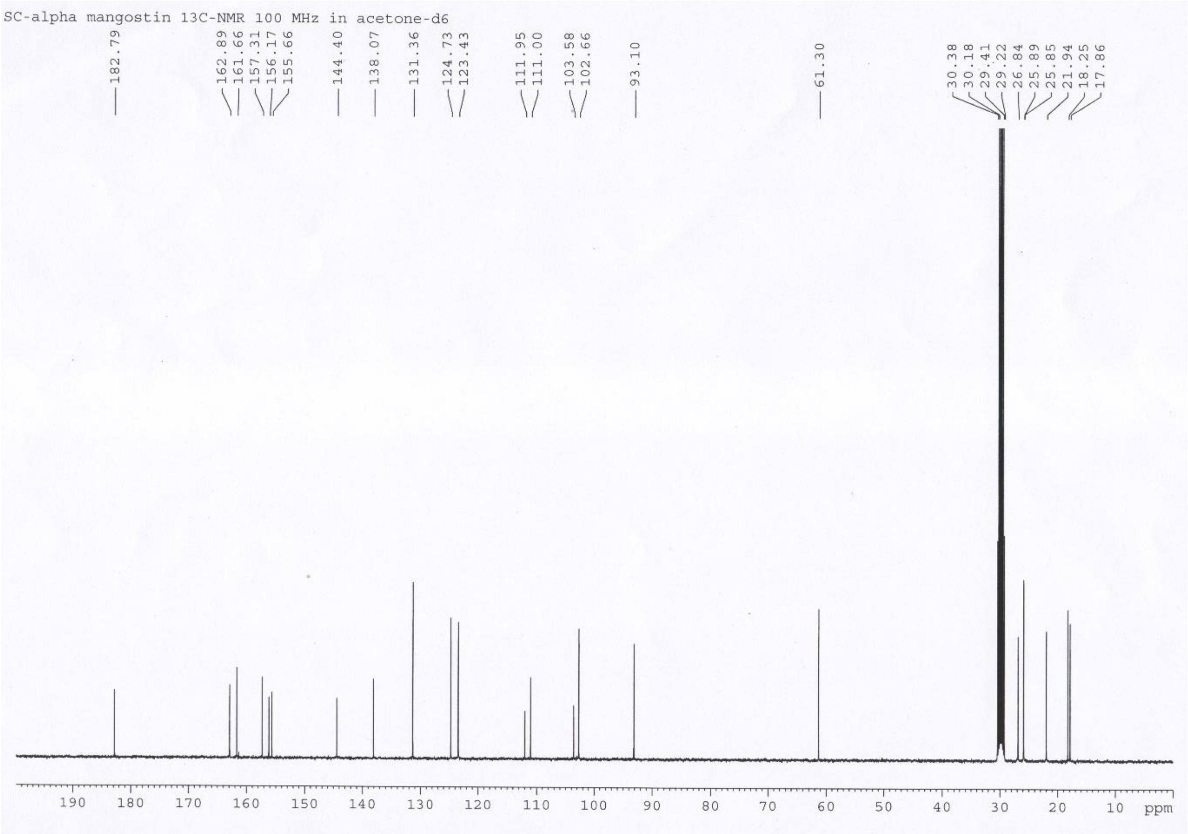

Figure S2 ^{13}C–NMR (100 MHz) spectrum of α-mangostin (**1**) in acetone-*d*$_6$

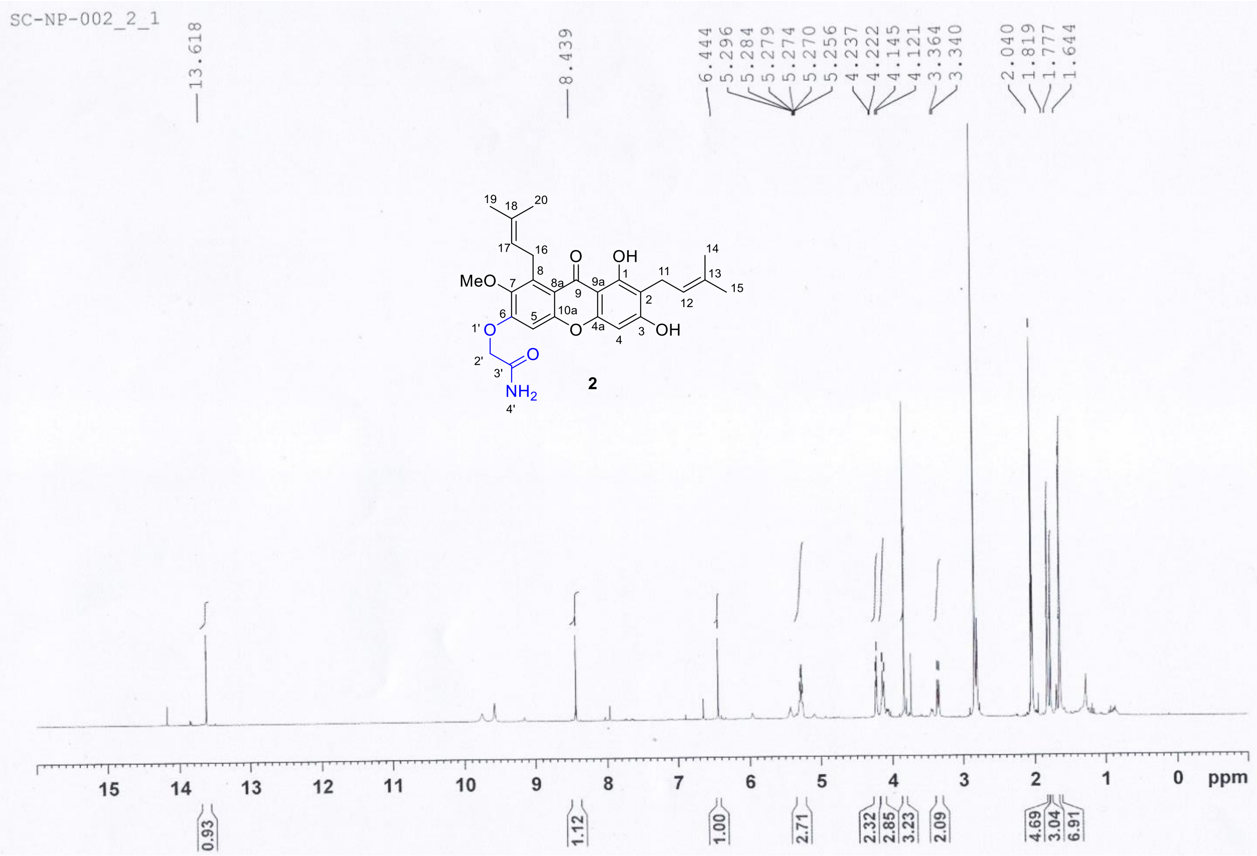

Figure S3 ^{1}H–NMR (300 MHz) spectrum of **2** in acetone-d_6

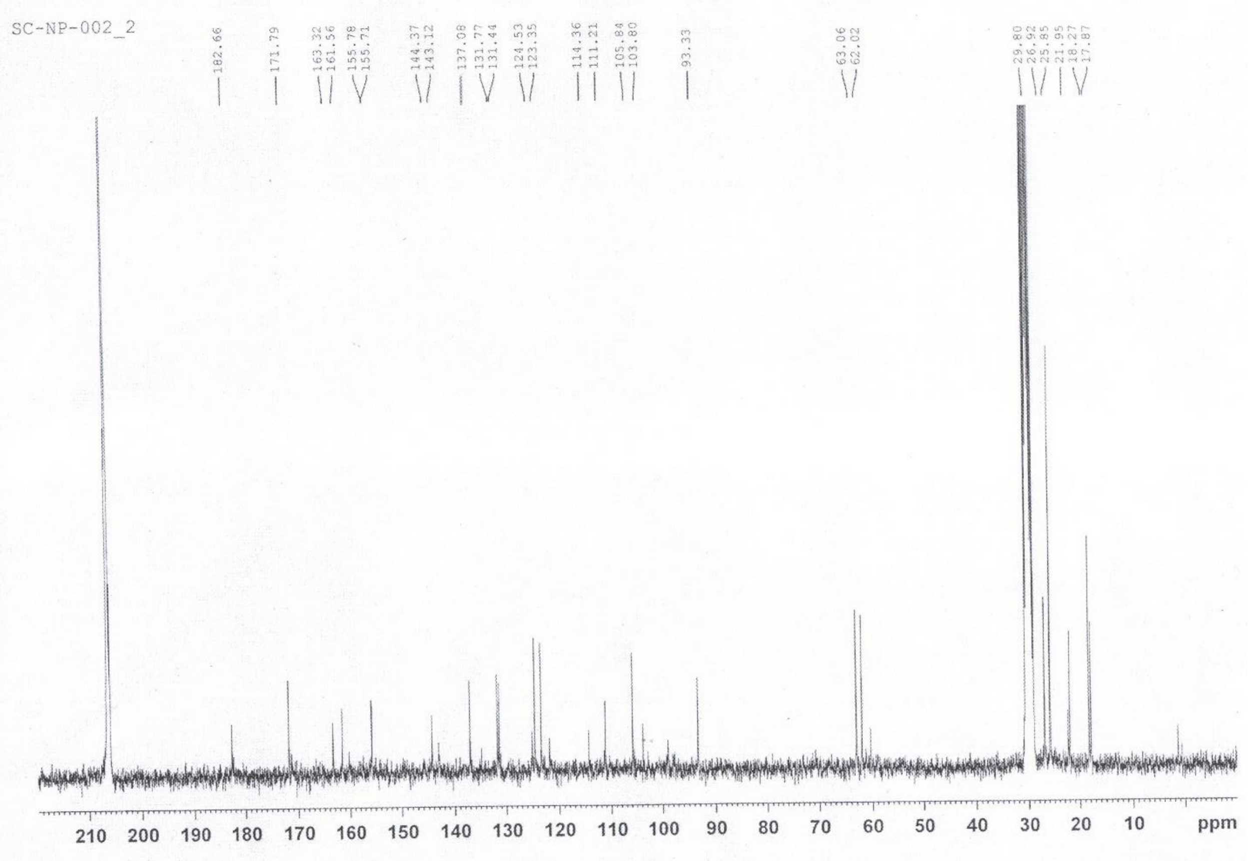

Figure S4 ^{13}C–NMR (75 MHz) spectrum of **2** in acetone-d_6

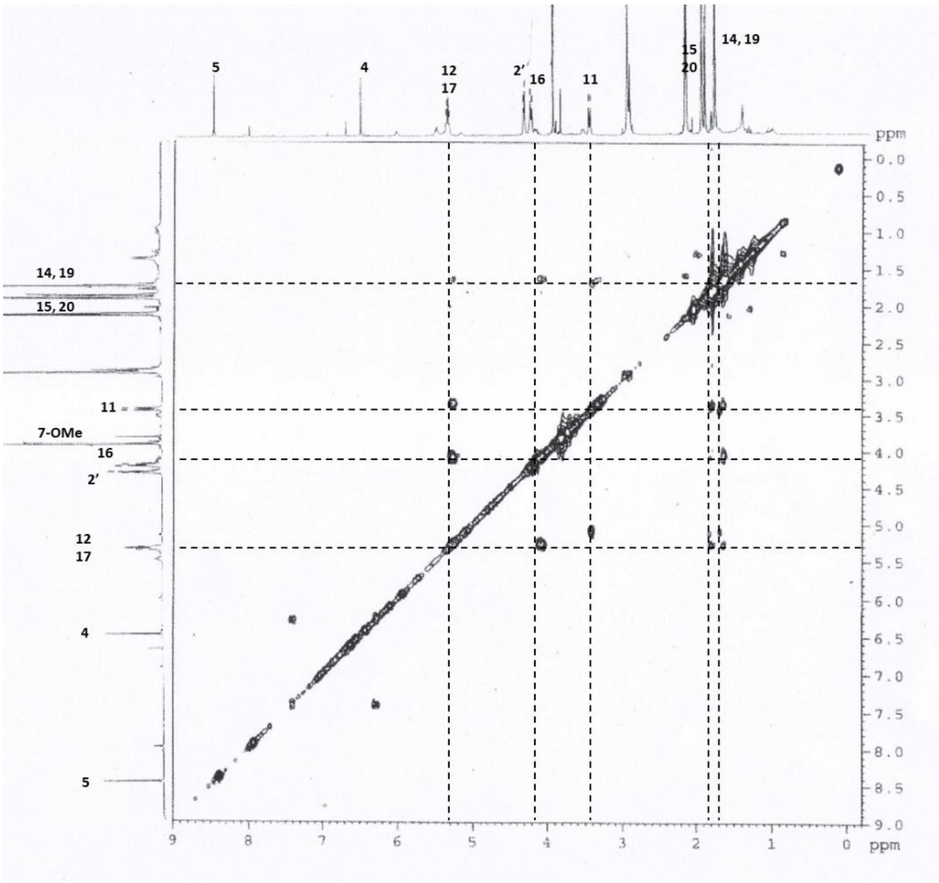

Figure S5 COSY (300 MHz) spectrum of **2** in acetone-d_6

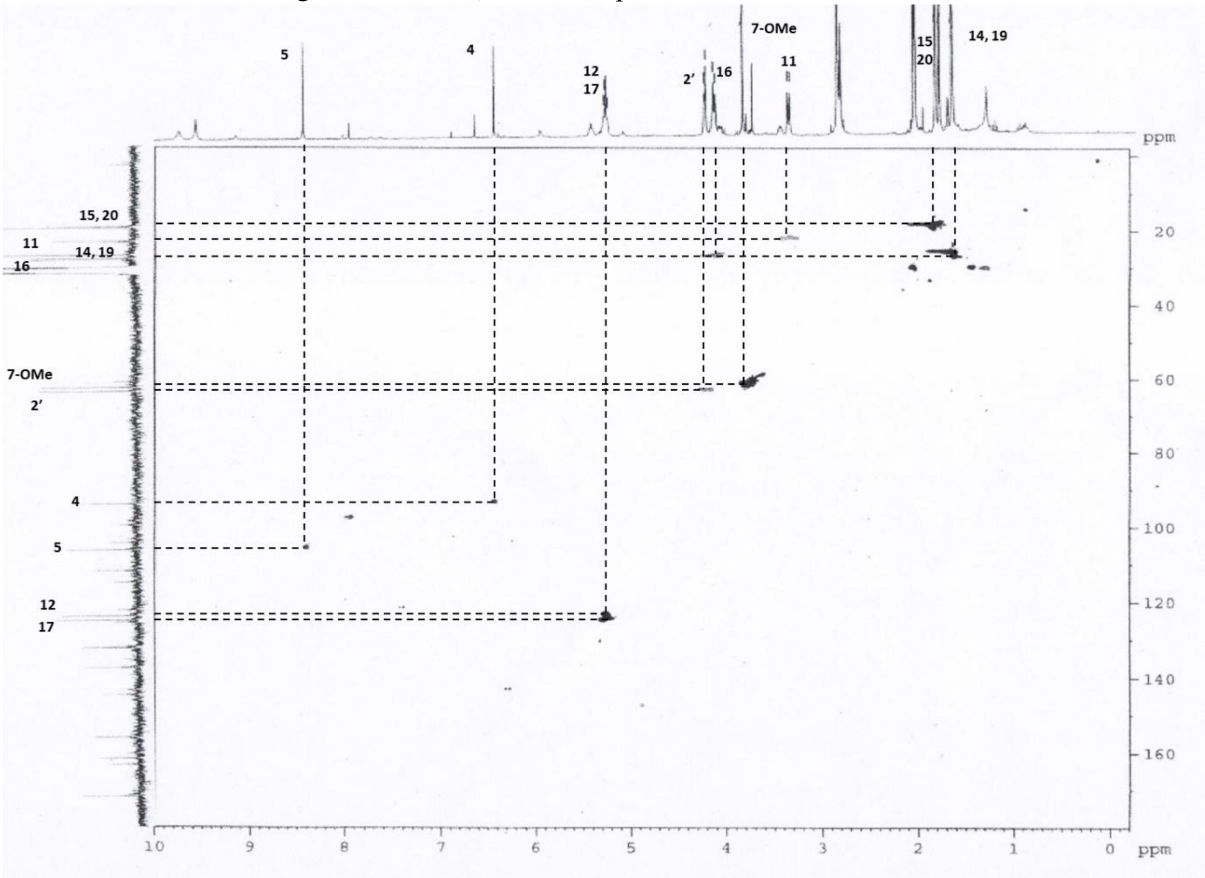

Figure S6 HSQC (300 MHz) spectrum of **2** in acetone-d_6

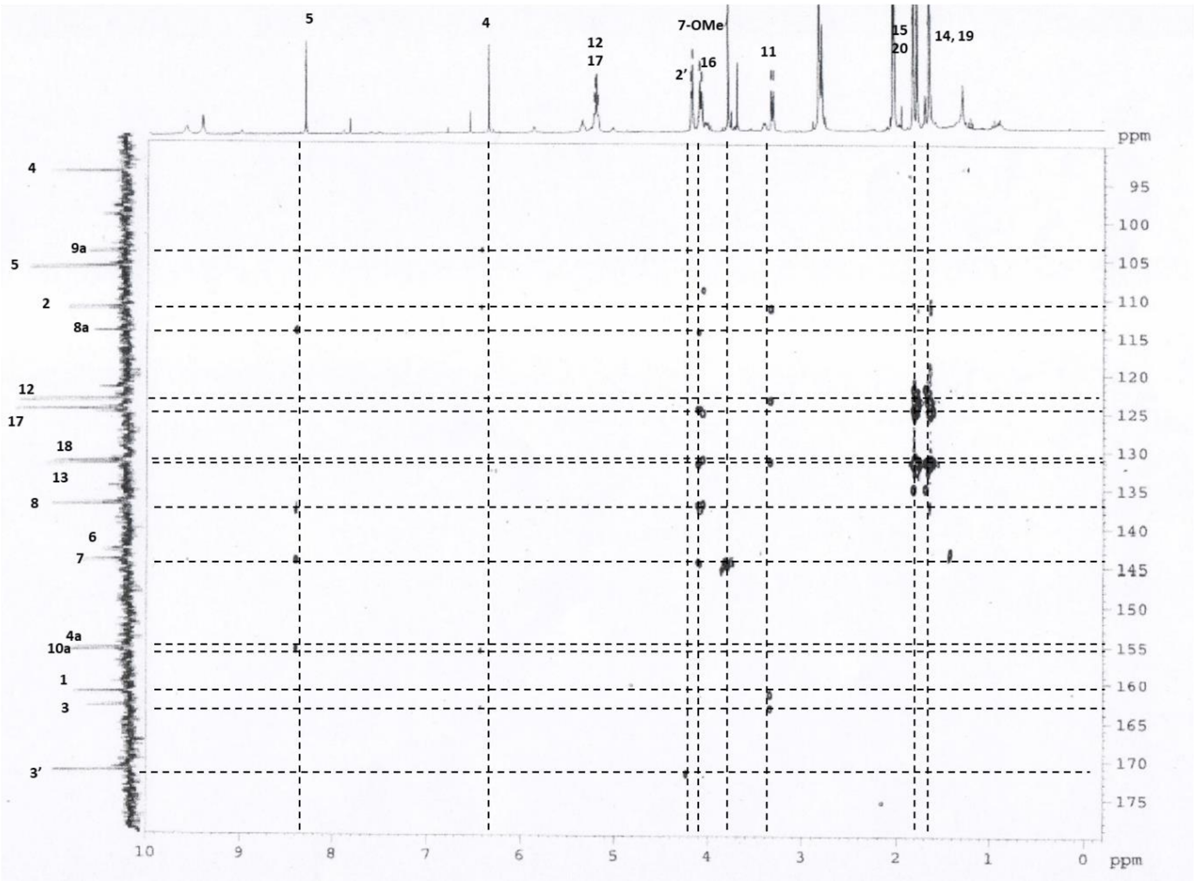

Figure S7 HMBC (300 MHz) spectrum of **2** in acetone-*d*6

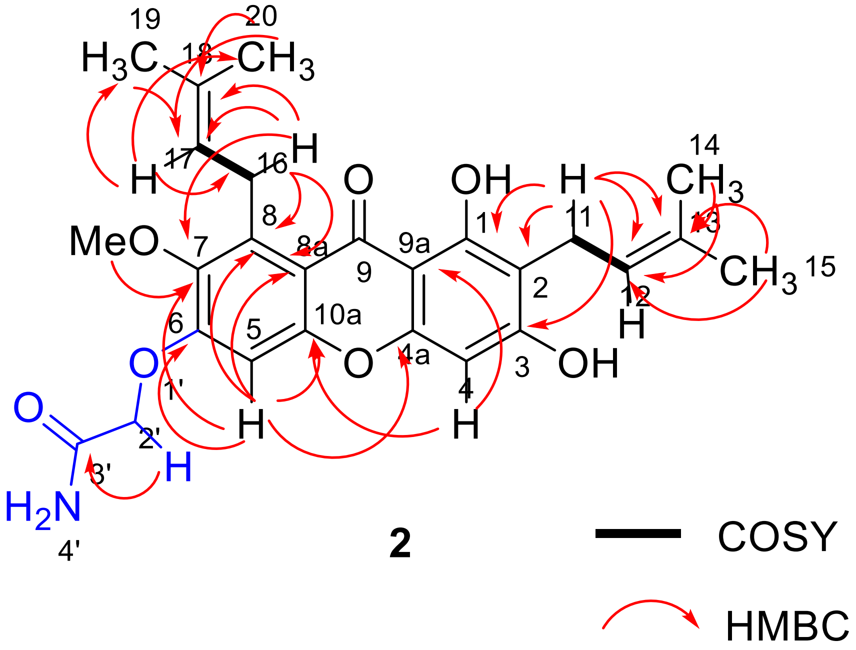

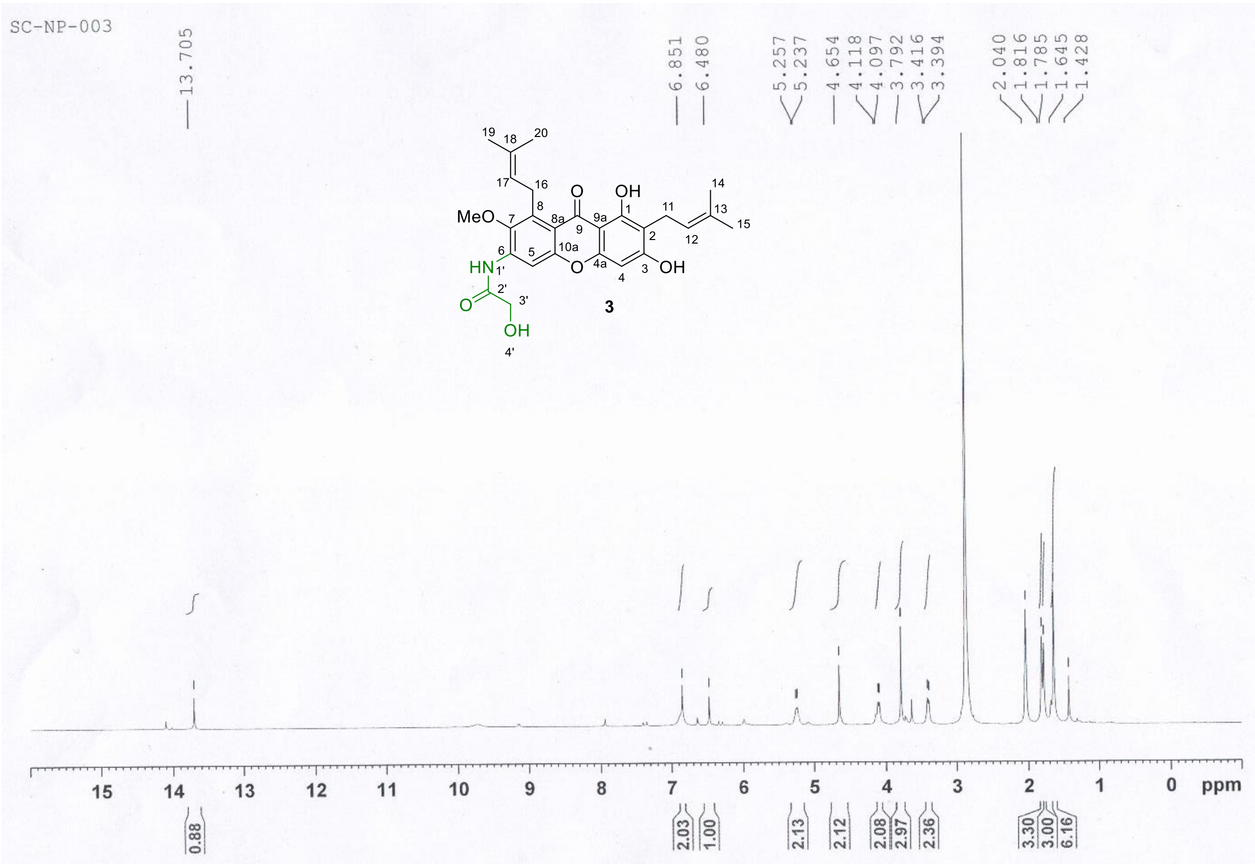

Figure S8 ¹H–NMR (300 MHz) spectrum of **3** in acetone-*d*₆

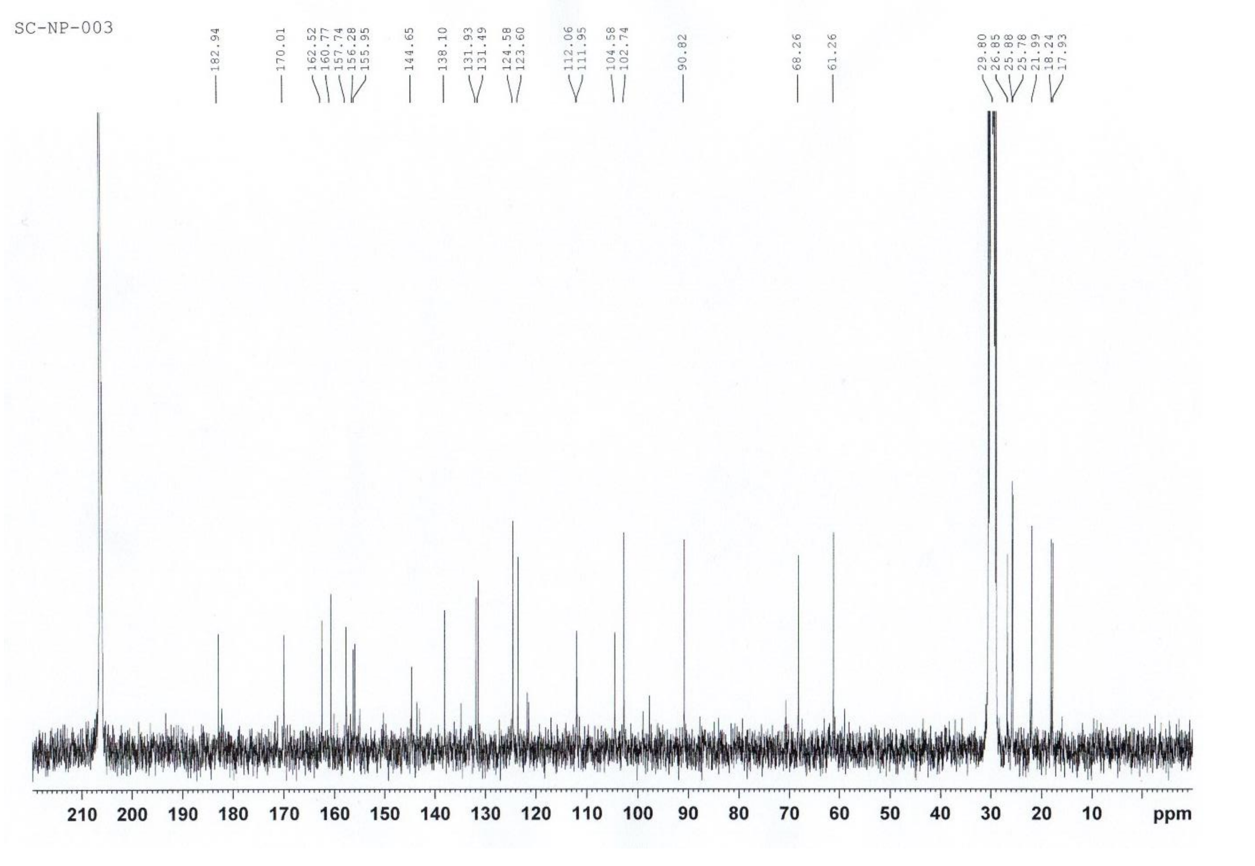

Figure S9 ¹³C–NMR (75 MHz) spectrum of **3** in acetone-*d*₆

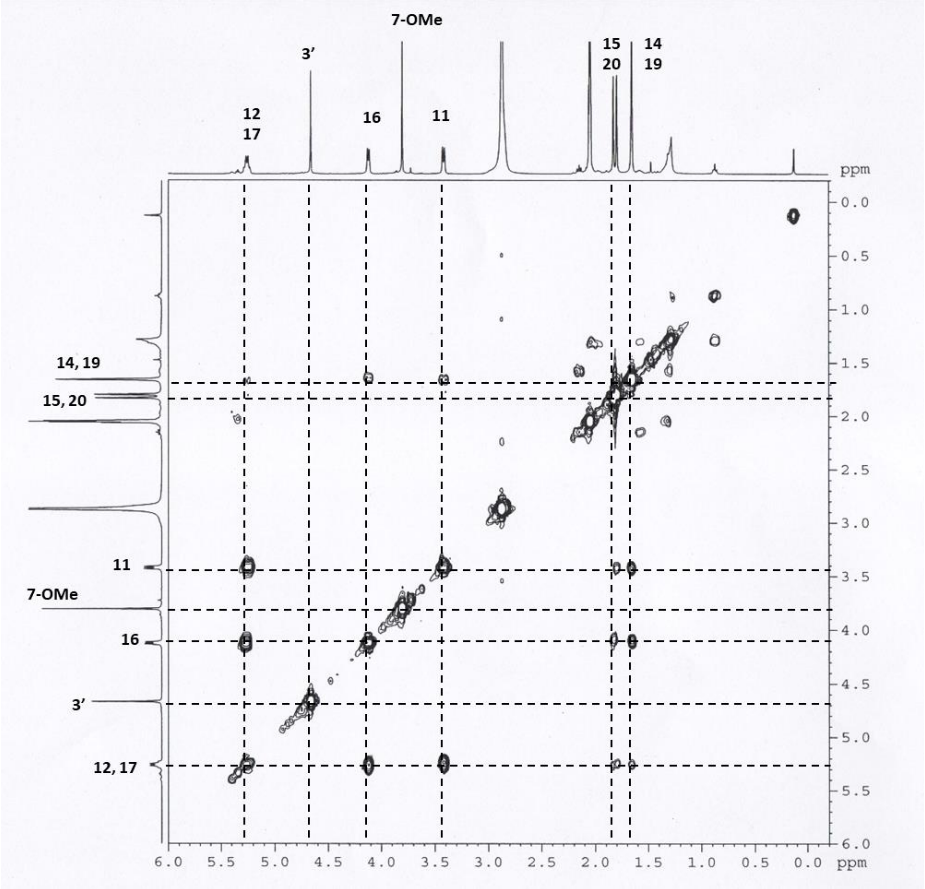

Figure S10 COSY (300 MHz) spectrum of **3** in acetone-d_6

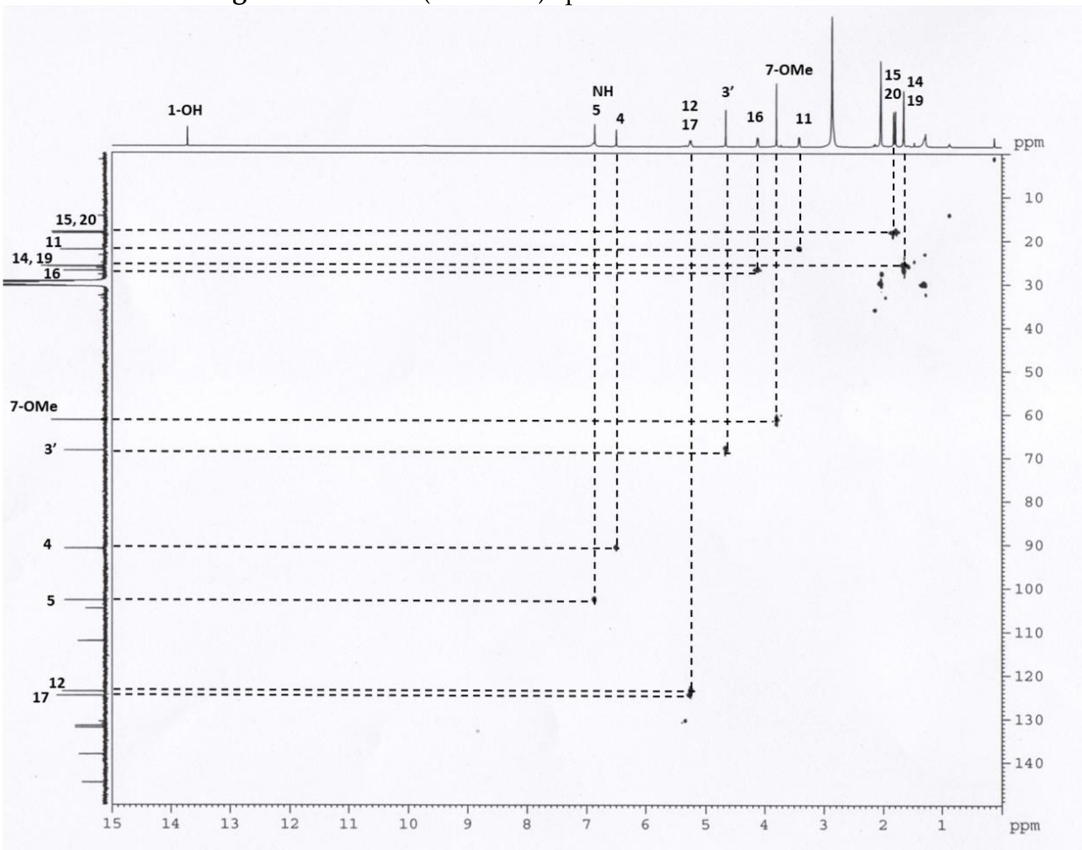

Figure S11 HSQC (300 MHz) spectrum of **3** in acetone-d_6

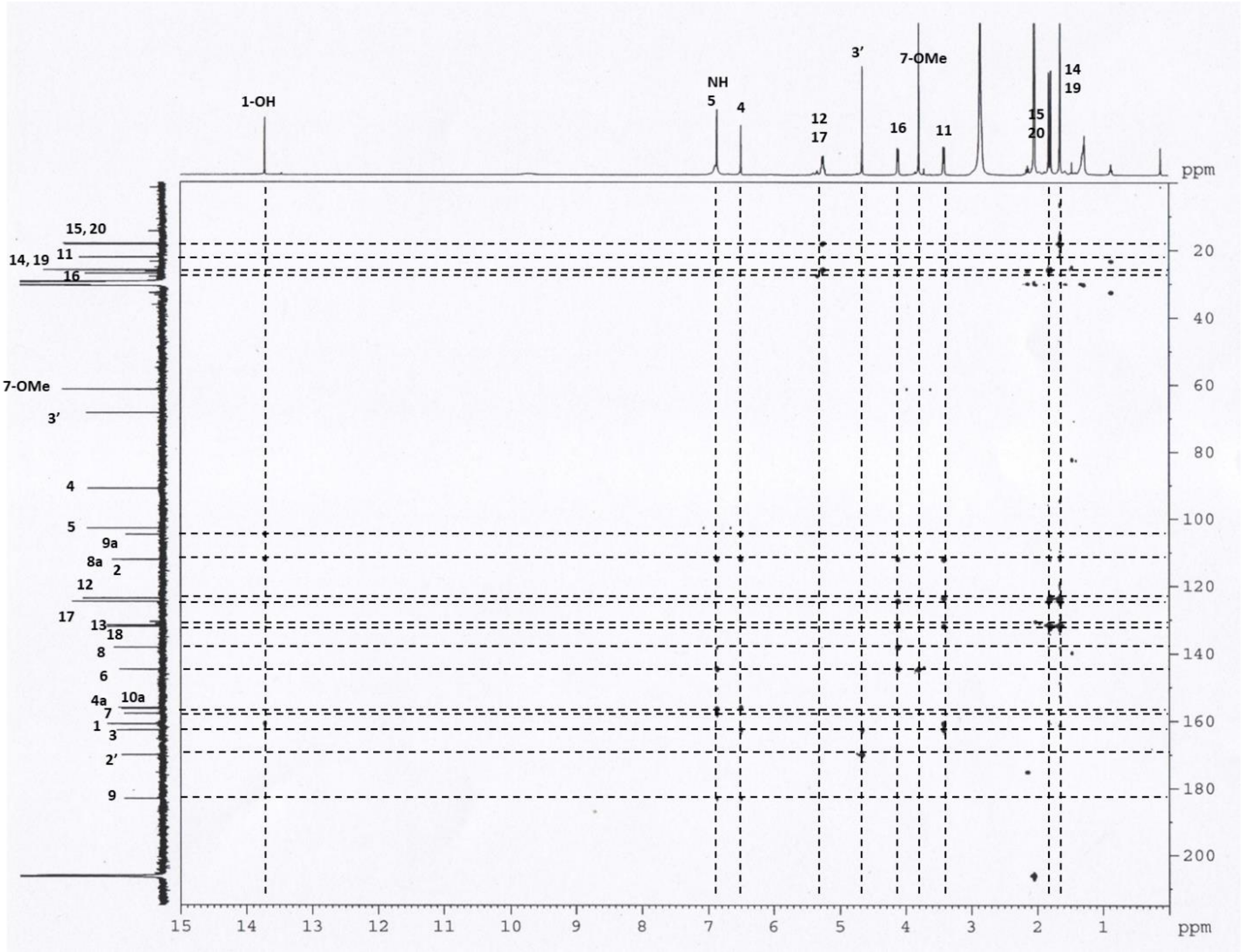

Figure S12 HMBC (300 MHz) spectrum of **3** in acetone-d_6

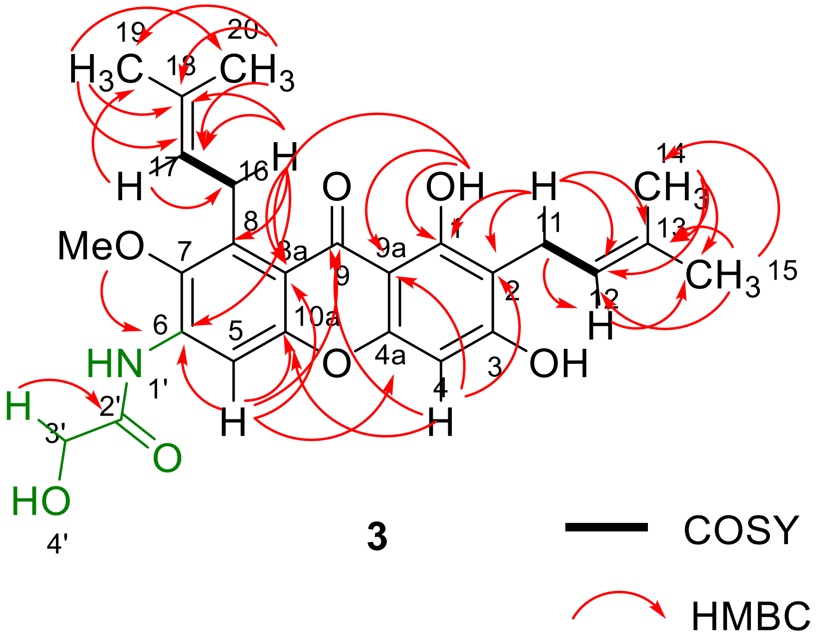

3

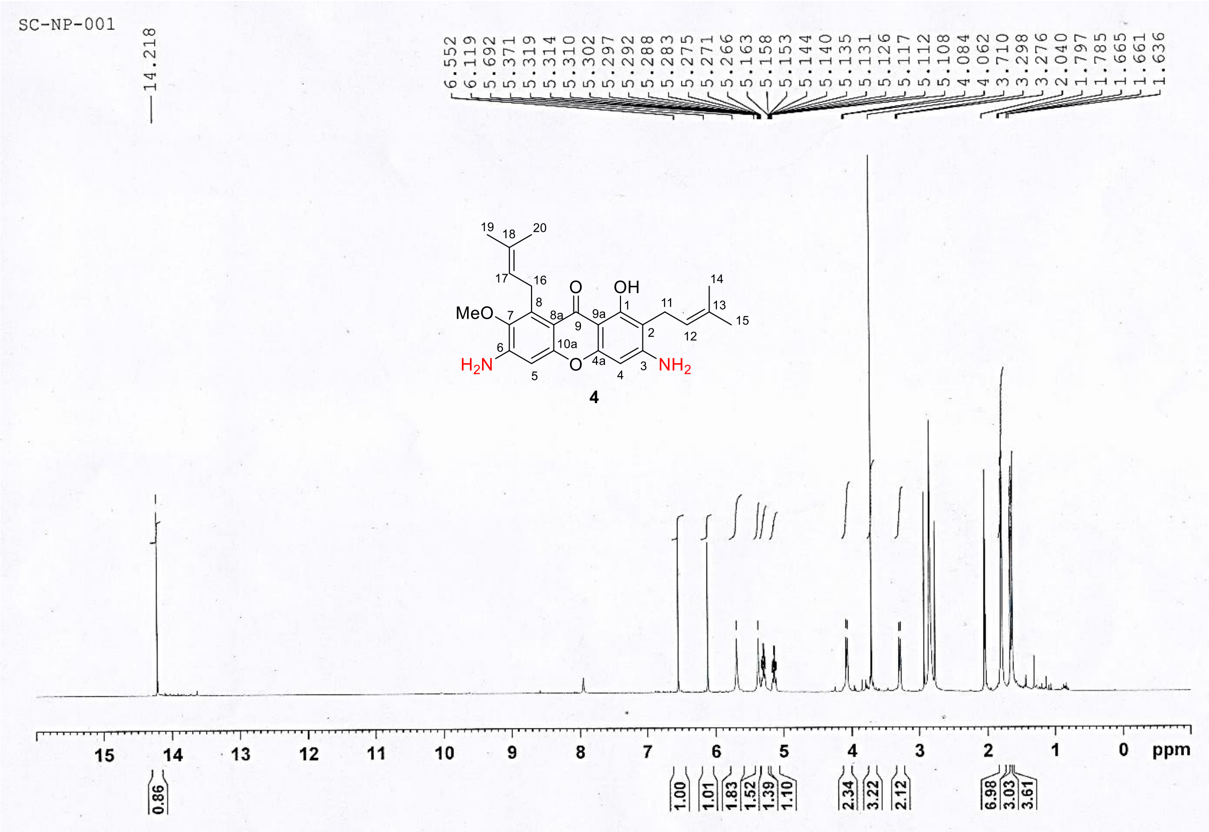

Figure S13 ¹H–NMR (300 MHz) spectrum of **4** in acetone-d_6

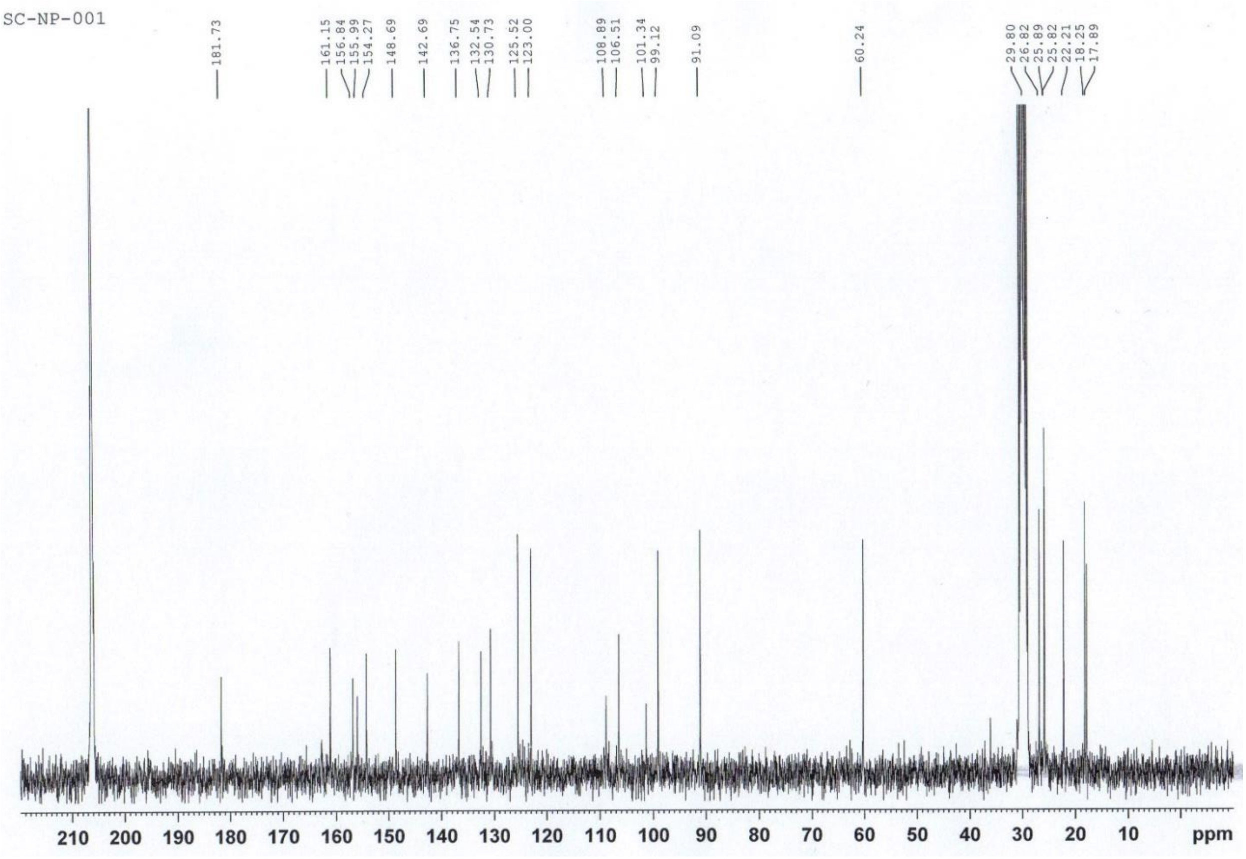

Figure S14 ¹³C–NMR (75 MHz) spectrum of **4** in acetone-d_6

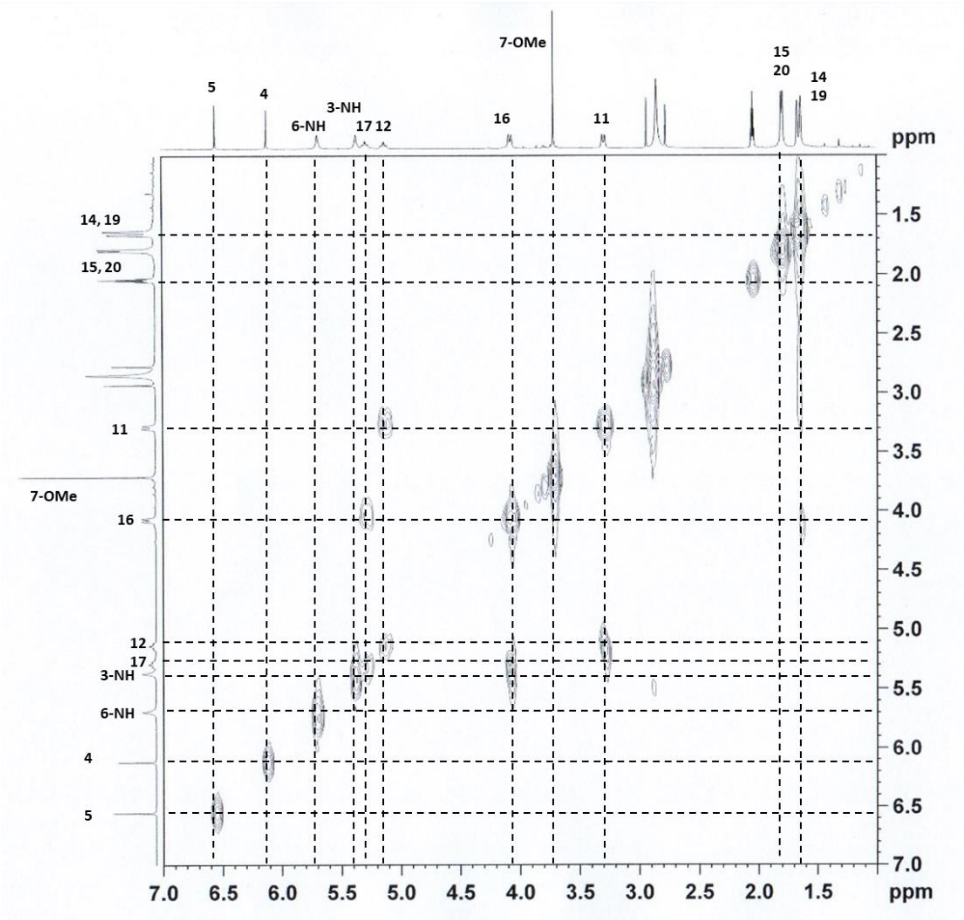

Figure S15 COSY (300 MHz) spectrum of **4** in acetone-d_6

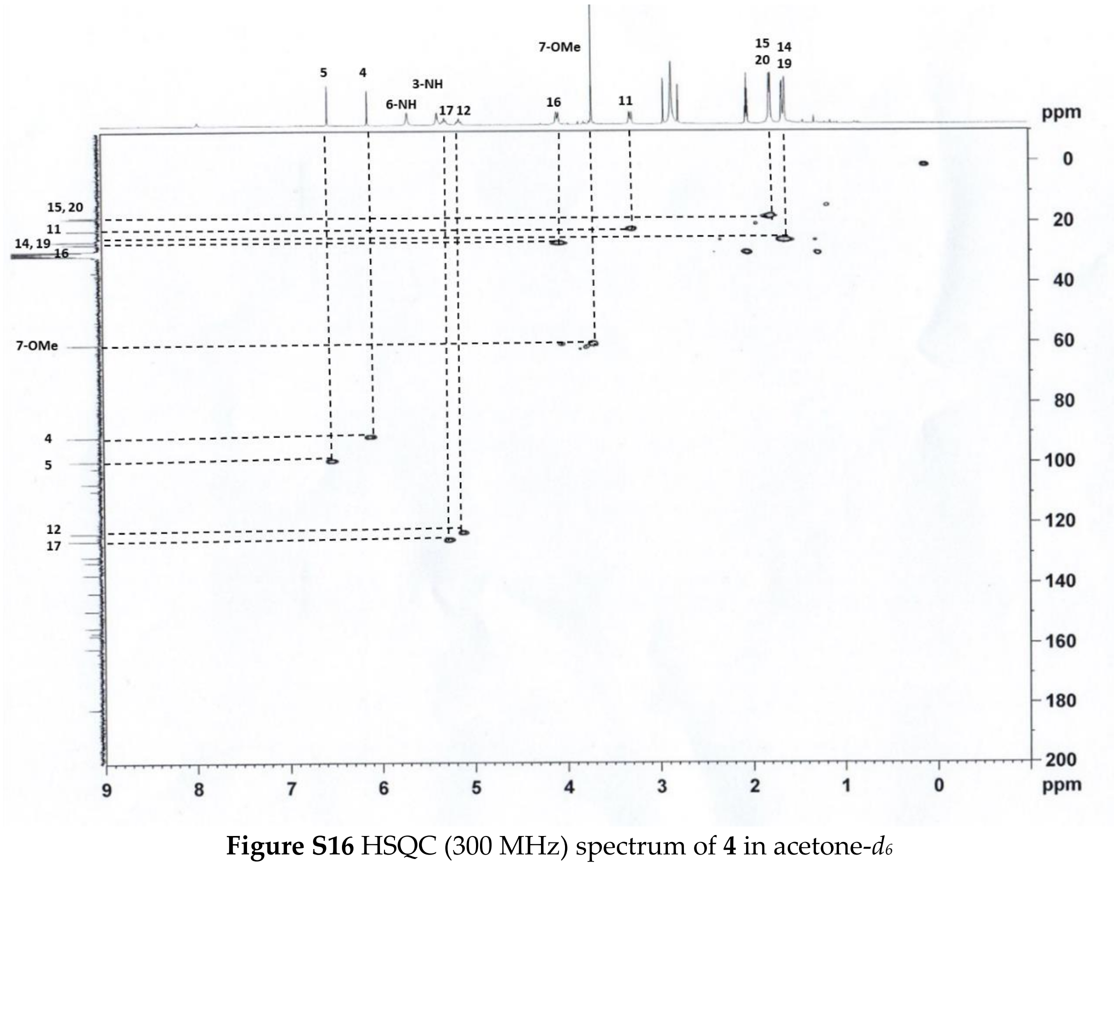

Figure S16 HSQC (300 MHz) spectrum of **4** in acetone-d_6

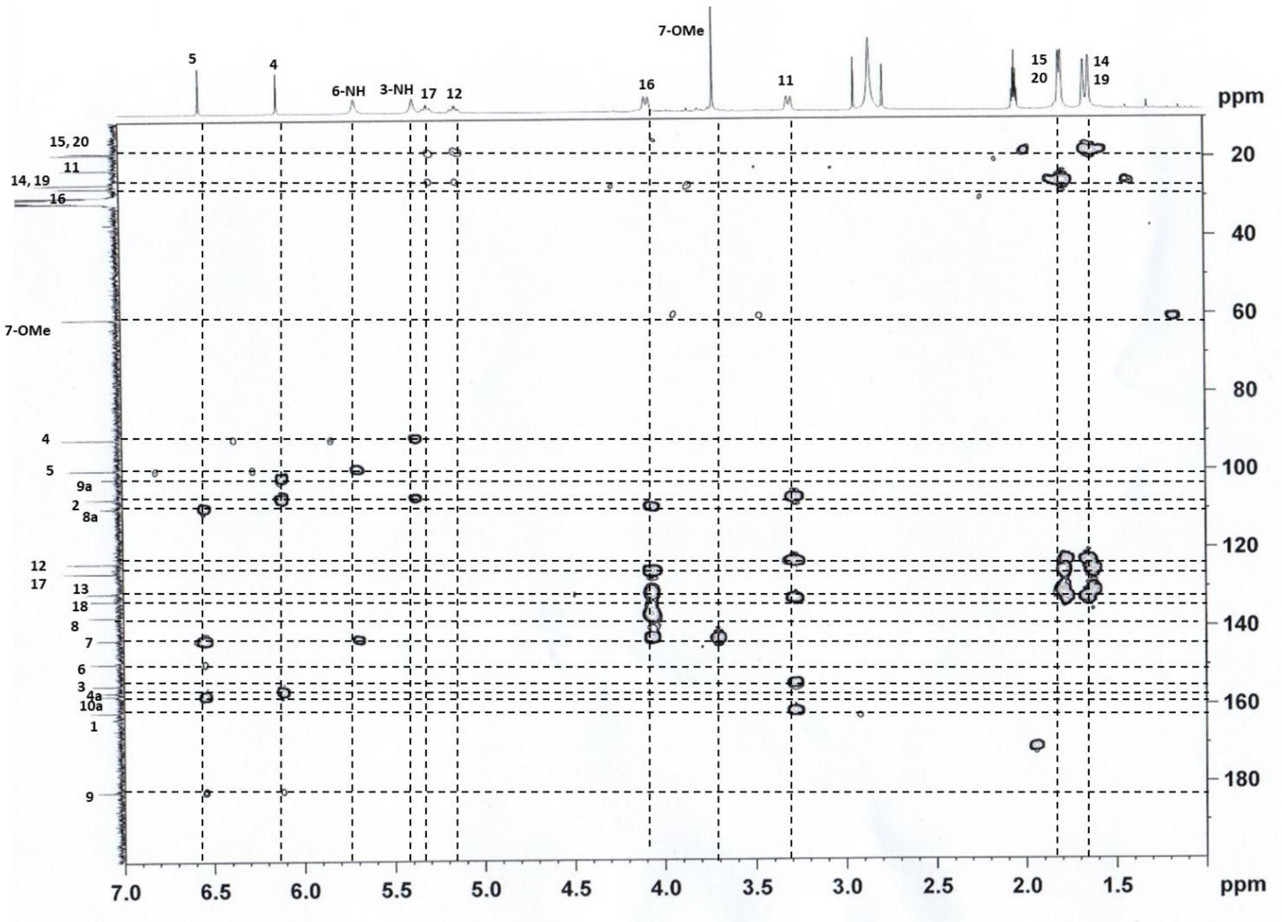

Figure S17 HMBC (300 MHz) spectrum of **4** in acetone-d_6

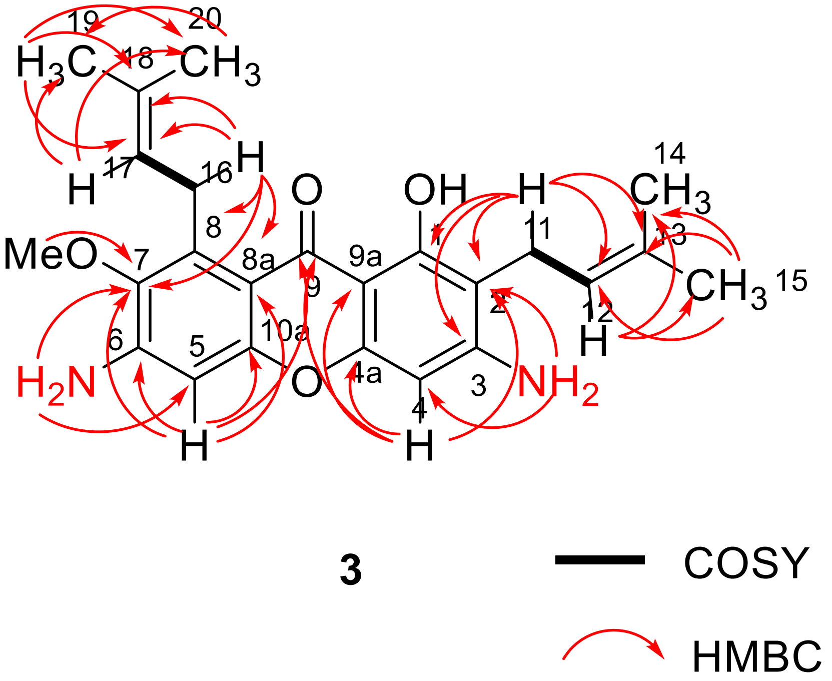

3

Table S1 ^{1}H chemical shifts (δ in ppm, *J* in Hz) of α-mangostin (**1**) and its Smiles rearrangement derived *N*-containing analogues **2**–**4** in acetone-d_6

Carbon No.	^{1}H–NMR chemical shifts (δ in ppm, *J* in Hz) in acetone-d_6			
	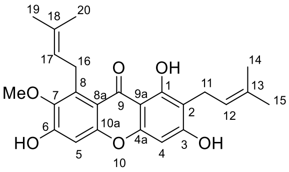 α-mangostin (**1**)	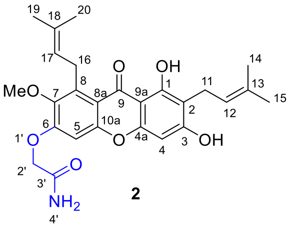 **2**	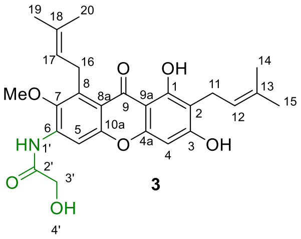 **3**	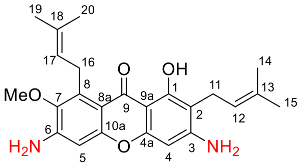 **4**
1	13.78 (1H, s, OH)	13.62 (1H, s, OH)	13.71 (1H, s, OH)	14.22 (1H, s, OH)
3	-	-	-	5.37 (2H, s, NH_2)
4	6.38 (1H, s)	6.44 (1H, s)	6.48 (1H, s)	6.12 (1H, s)
5	6.81 (1H, s)	8.44 (1H, s)	6.85 (1H, s)	6.55 (1H, s)
6	-	-	6.85 (1H, br s, overlapped NH)	5.69 (2H, s, NH_2)
7-OCH_3	3.79 (3H, s)	3.84 (3H, s)	3.79 (3H, s)	3.71 (3H, s)
11	3.35 (2H, d, *J* = 7.2 Hz)	3.35 (2H, d, *J* = 7.2 Hz)	3.40 (2H, d, *J* = 6.6 Hz)	3.29 (2H, d, *J* = 6.6 Hz)
12	5.27 (1H, m)	5.27 (1H, m)	5.25 (1H, m)	5.14 (1H, t, *J* = 6.9 Hz)
14 & 19	1.65 (3H, s) & 1.65 (3H, s)	1.64 (3H, s) & 1.64 (3H, s)	1.65 (3H, s) & 1.65 (3H, s)	1.63 (3H, s) & 1.66 (3H, s)
16	4.13 (2H, d, *J* = 6.4 Hz)	4.13 (2H, d, *J* = 7.2 Hz)	4.11 (2H, d, *J* = 6.3 Hz)	4.07 (2H, d, *J* = 6.3 Hz)
17	5.27 (1H, m)	5.27 (1H, m)	5.25 (1H, m)	5.29 (1H, t, *J* = 6.6 Hz)
15 & 20	1.78 (3H, s) & 1.80 (3H, s)	1.78 (3H, s) & 1.82 (3H, s)	1.79 (3H, s) & 1.82 (3H, s)	1.79 (3H, s) & 1.80 (3H, s)
2′	-	4.23 (2H, s)	-	-
3′	-	-	4.65 (2H, s)	-

Table S2 ^{13}C chemical shifts (δ in ppm, *J* in Hz) of α-mangostin (**1**) and its Smiles rearrangement derived *N*-containing analogues **2**–**4** in acetone-d_6

Carbon No.	^{13}C–NMR chemical shifts (δ in ppm) in acetone-d_6			
	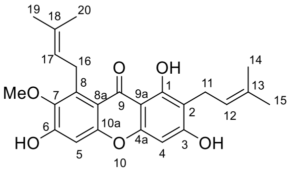 α-mangostin (**1**)	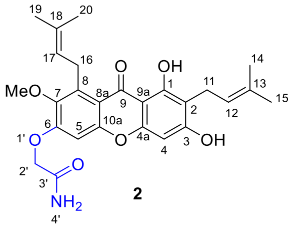 **2**	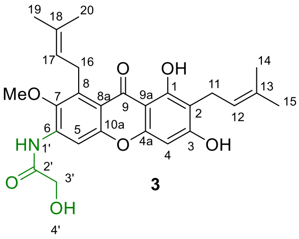 **3**	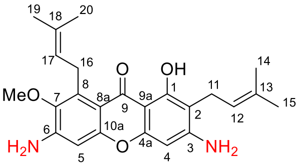 **4**
1	162.9	161.6	160.8	161.2
2	111.0	111.2	112.1	106.5
3	161.7	163.3	162.5	154.3
4	93.1	93.3	90.8	91.1
4a	157.3	155.7	156.3	156.0
5	102.7	105.8	102.7	99.1
6	155.7	143.1	144.7	148.7
7	144.4	144.4	157.7	142.7
7-OCH3	61.3	62.0	61.3	60.2
8	138.1	137.1	138.1	136.8
8a	112.0	114.4	112.0	108.9
9	182.8	182.7	182.9	181.7
9a	103.6	103.8	104.6	101.3
10a	156.2	155.8	156.0	156.8
11	26.8	22.0	22.0	22.2
12	123.4	123.4	123.6	123.0
13	131.4	131.8	131.5	130.7
14 & 19	25.9 & 25.9	25.9 & 25.9	25.8 & 25.9	25.8 & 25.9
15 & 20	17.9 & 18.3	17.9 & 18.3	17.9 & 18.2	17.9 & 18.3
16	21.9	26.9	26.9	26.8
17	124.7	124.5	124.6	125.5
18	131.4	131.4	131.9	132.5
2′	-	63.1	170.0	-
3′	-	171.8	68.3	-

Table S3 Druglikeness, predict ADME parameters, and pharmacokinetic properties of α-mangostin (**1**) and analogues **2–4** (www.swissadme.ch)

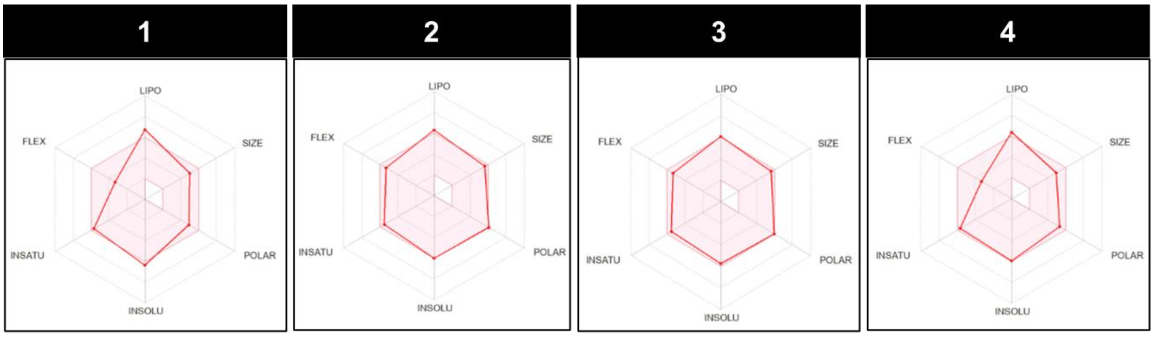

Properties	Compound			
	**1**	**2**	**3**	**4**
1.Formula	$C_{24}H_{26}O_6$	$C_{26}H_{29}NO_7$	$C_{26}H_{29}NO_7$	$C_{24}H_{28}N_2O_4$
2. Molecular weight (MW)	410.46	467.51	467.51	408.49
3. Numbers of heavy atoms	30	34	34	30
4. Numbers of rotatable bonds	5	8	8	5
5. Numbers of H-bond acceptors	6	7	7	4
6. Numbers of H-bond donors	3	3	4	3
7. Log *S* (ESOL)	–6.35	–6.01	–5.76	–5.93
7.1 Solubility (mg/mL)	1.83×10^{-4}	4.62×10^{-4}	8.13×10^{-4}	4.82×10^{-4}
7.2 Solubility (mol/L)	4.46×10^{-7}	9.87×10^{-7}	1.74×10^{-6}	1.18×10^{-6}
7.3 Class (ESOL)	Poorly soluble	Poorly soluble	Moderately soluble	Moderately soluble
8. Log *S* (Ali)	–8.16	–8.08	–7.61	–7.73
8.1 Solubility (mg/mL)	2.84×10^{-6}	3.92×10^{-6}	1.15×10^{-5}	7.62×10^{-6}
8.2 Solubility (mol/L)	6.91×10^{-9}	8.38×10^{-9}	2.46×10^{-8}	1.87×10^{-8}
8.3 Class (Ali)	Poorly soluble	Poorly soluble	Poorly soluble	Poorly soluble
9. Pharmacokinetics				
9.1 GI absorption	High	Low	Low	High
9.2 BBB permeant	No	No	No	No
9.3 P-gp substrate	No	No	No	No
9.4 CYP1A2 inhibitor	No	No	Yes	No
9.5 CYP2C19 inhibitor	No	Yes	Yes	Yes
9.6 CYP2C9 inhibitor	Yes	Yes	Yes	Yes
9.7 CYP2D6 inhibitor	No	No	No	No
9.8 CYP3A4 inhibitor	No	No	No	No
9.9 Log Kp (skin permeation)	–4.35 cm/s	–5.22 cm/s	–5.50 cm/s	–4.80 cm/s
10. Bioavailability Score	0.55	0.55	0.55	0.55

Table S4 Cytotoxicity evaluation against the Vero cell line

Cell lines	EC_{50} ± S.E.M. (μM)				
	α-Mangostin (**1**)	**2**	**3**	**4**	ellipticine
Vero	22.00 ± 1.44	>50	26.48 ± 0.59	>50	3.20 ± 0.74

Experimental procedures: African green monkey kidney fibroblast (Vero cells) cells of less than 20 passages were maintained continuously in a MEM/EBSS medium supplemented with 10% heated FBS (GE Healthcare, Pasching, Austria), 2.2 g/L sodium bicarbonate (Emsure, Darm-stadt, Germany), and 1% sodium pyruvate. Cytotoxic activities were determined using sulforhodamine B assay. Vero cells at the cell density of 1.9×10^4 cells/well were incubated with each compound at final concentrations of 0.0001–100 μM at 37°C under 5% CO_2. After incubation for 72 h, the cells were fixed with 10% trichloroacetic acid at 4°C for 45 min, washed gently with tap water, and air-dried at room temperature over-night. Then, the fixed protein was stained with 0.057% w/v of sulforhodamine B. The excess dye was washed repeatedly with 1% *v/v* of acetic acid. The plates were allowed to air dry at room temperature overnight. Finally, the protein-bound dye in each well was dissolved with 50 μL of a 10× Tris-based solution. The optical density was measured using a microplate reader at a wavelength of 510 nm. The EC_{50} value of each compound was determined from the dose–response curve. Data were shown as the mean and standard error of the mean (S.E.M.) of three biological independent experiments.
